# Recent Advances in the Surgical Management of Thyroid Cancer

**DOI:** 10.3390/curroncol30050361

**Published:** 2023-05-05

**Authors:** Boris Scheller, Dorian Culié, Gilles Poissonnet, Olivier Dassonville, Grégoire D’Andréa, Alexandre Bozec

**Affiliations:** 1 Face and Neck University Institute, 31 Av. de Valombrose, 06103 Nice, France; 2 Antoine Lacassagne Center, 33 Av. de Valombrose, 06189 Nice, France; 3 University Hospital Center of Nice, 30 Av. de la Voie Romaine, 06000 Nice, France; 4 Faculty of Medecine, Côte D’Azur University, 28 Av. Valrose, 06108 Nice, France

**Keywords:** thyroid cancer, papillary thyroid carcinoma, surgery

## Abstract

A growing incidence of differentiated thyroid cancer (DTC) has been reported in most developed countries, corresponding mainly to incidentally discovered small papillary thyroid carcinomas. Given the excellent prognosis of most patients with DTC, optimal therapeutic management, minimizing complications, and preserving patient quality of life are essential. Thyroid surgery has a central role in both the diagnosis, staging, and treatment of patients with DTC. Thyroid surgery should be integrated into the global and multidisciplinary management of patients with DTC. However, the optimal surgical management of DTC patients is still controversial. In this review article, we discuss the recent advances and current debates in DTC surgery, including preoperative molecular testing, risk stratification, the extent of thyroid surgery, innovative surgical tools, and new surgical approaches.

## 1. Introduction

An increasing incidence of thyroid nodules has been reported worldwide during the past few decades, corresponding mostly to incidental findings due to a continuing growth in the use of medical imaging procedures [1,2,3]. The incidence of differentiated thyroid cancer (DTC, i.e., papillary and follicular thyroid carcinomas) has also increased [1,3], consisting mainly of small papillary thyroid carcinomas (PTC) that are associated with very high survival rates [4]. A rising incidence of autoimmune thyroid diseases such as Hashimoto’s thyroiditis has also been reported, along with a potential association with the risk of PTC [3]. Whereas the conventional treatment of DTC involved total thyroidectomy (TT) with radioiodine (RAI) adjuvant therapy, the growing incidence of low-risk DTC over the last decades has led to a re-evaluation of the traditional therapeutic approach to DTC. 

The modifications made in the AJCC/TNM staging system and the development of the ATA (American Thyroid Association) risk-stratification system for prediction of disease recurrence allow an accurate evaluation of the tumor recurrence risk for each patient [4,5]. Progressively, a more personalized therapeutic approach has been developed according to the individual risk of recurrence and treatment failure [4]. Given the excellent prognosis of this disease, reduction of treatment-related morbidity and preservation of quality of life (QoL) are essential for DTC patients. Innovative technological tools and surgical approaches have been developed to further reduce thyroidectomy-related complications [4]. 

Despite several international therapeutic guidelines, the optimal surgical management of DTC is still controversial, particularly regarding the indications for surgery, the extent of thyroidectomy, and the role of prophylactic neck dissection [4,6,7,8]. 

The aim of this review is to discuss the optimal surgical management of patients with DTC, highlighting the recent advances and current debates in the field of thyroid oncologic surgery.

## 2. Preoperative Evaluation

### 2.1. Neck Ultrasonography

Neck ultrasonography (US) is a cornerstone in the preoperative assessment of thyroid nodules [2]. Hypoechogenicity, non-oval shape, irregular margins, and microcalcifications are the main criteria used to define the risk of malignancy [2,4,9]. US findings are summarized in the EU-TIRADS classification, which defines five categories of risk, with the EU-TIRADS 5 score being associated with the highest risk of malignancy (26 to 87%) [9]. Along with nodule size, EU-TIRADS classification is used to indicate a fine needle aspiration biopsy (FNAB) of suspicious thyroid nodules [9]. Briefly, FNAB is indicated for EU-TIRADS 5 nodules >10 mm, EU-TIRADS 4 nodules >15 mm and EU-TIRADS 3 nodules >20 mm [2,9]. 

Besides evaluating the risk of malignancy of each thyroid nodule and guiding FNAB, neck US is essential in the preoperative staging of suspicious / malignant thyroid nodules [2,10]. In the preoperative setting, US should assess precisely the presence of an extrathyroidal extension (ETE) of the tumor and particularly the risk of involvement of the strap muscles, trachea, and recurrent laryngeal nerve (RLN) [2,10]. Moreover, neck US should evaluate the status of the cervical lymph nodes in the central (level VI) and lateral compartments (levels II to IV) [10]. FNAB of cervical lymph nodes deemed to be suspicious should be performed under US guidance if they have a short diameter ≥8–10 mm and if confirmation of malignancy would change surgical management [4,6]. 

### 2.2. Fine Needle Aspiration Biopsy

The most reliable diagnostic procedure for thyroid nodules is FNAB, which provides an accurate diagnosis (benign vs. malignant) for most patients [2,4,6]. However, in 10–25% of cases, thyroid nodules are cytologically diagnosed as indeterminate and will potentially require thyroid surgery only for diagnostic purposes [11]. Indeterminate thyroid nodules are those that, according to the Bethesda system for reporting thyroid cytopathology, are classified as Bethesda class III (“atypia of undetermined significance” or “follicular lesions of undetermined significance”) or IV (“follicular neoplasm or suspicious for a follicular or Hürthle cell neoplasm”) and are associated with a malignancy risk of 5% to 15% and 15% to 30%, respectively [11]. 

In common clinical practice, repeat FNAB is recommended for Bethesda class III nodules and surgical lobectomy for Bethesda class IV nodules [4,5]. However, since a small proportion of cytologically indeterminate nodules prove malignant in the surgical pathology report, thyroid surgery is considered unnecessary in a considerable number of patients [2,11]. The need to reduce these potentially avoidable diagnostic thyroidectomies has paved the way for the development of molecular testing for thyroid nodules. 

### 2.3. Thyroid Nodule Molecular Testing

Molecular testing of the FNAB samples is a recent approach that could reduce the need for diagnostic surgery [2,12]. The tests developed for this purpose over the past 10 years are based on three main molecular approaches: testing for somatic mutations, gene expression profiling, and microRNA (miRNA)-based classifiers [12,13,14,15,16]. The performance of molecular tests depends on the institutional prevalence of malignancy in each cytological category, as this prevalence will affect the positive and negative predictive values of the molecular test used [17]. Ideally, a rule-out test would have a high negative predictive value (NPV), as a benign cytological diagnosis, and a rule-in test would have a high positive predictive value (PPV), as a malignant cytological diagnosis [17]. ThyroSeq version 3(v3) and Afirma multigene classifier tests became available for clinical use in 2017 [13,14,15,18]. 

ThyroSeq v3 involves targeted next-generation sequencing analysis of 112 cancer-related genes for point mutations, gene fusions, copy number alterations, or abnormal gene expression (such as BRAF and RAS and PAX8—PPARG/RET—PTC) [13,14]. In a prospective study evaluating 286 cytologically indeterminate nodules, including 257 (90%) nodules with an informative molecular test, it demonstrated a 94% sensitivity and 82% specificity in Bethesda III and IV nodules combined [14]. With a cancer prevalence of 28%, the NPV was 97% and the PPV was 66%. The authors concluded that the test could eliminate the need for diagnostic surgery in up to 61% of patients with indeterminate nodules [14]. 

The current version of the Afirma genomic sequencing classifier (GSC) combines next-generation RNA sequencing with machine learning algorithms [15]. In a recent study including 183 patients, this GSC had a sensitivity of 91% and a specificity of 68%. At 24% cancer prevalence, the NPV was 96% and the PPV was 47%. This high accuracy for identifying benign thyroid nodules has the potential to increase the number of patients who can safely avoid unnecessary diagnostic surgery [18,19]. 

Other molecular tests, notably micro-RNA (miRNA) classifier tests such as the ThyraMIR and the RosettaGX Reveal (Rosetta Genomics) tests, have been developed and showed encouraging preliminary results [20,21]. 

The main characteristics of the most widely used molecular tests to characterize thyroid nodules are summarized in Table 1. Meaningful comparison of these molecular tests in terms of diagnostic performance is extremely difficult since currently available data come from studies that differ significantly in patient selection criteria, sample size, malignancy rate, study design, and applied reference [21]. Furthermore, the high cost of these molecular tests has greatly limited their use, even in many developed countries [21]. However, when hypothetical modeling was used to compare diagnostic surgery vs. molecular testing for the management of indeterminate nodules, both Thyroseq v3 and Afirma GSC proved to be considerably more cost-effective than diagnostic lobectomy, and the Thyroseq v3 was more cost-effective than the Afirma GSC [12]. The cost-effectiveness of molecular tests vs. thyroid surgery is still controversial and depends on the healthcare system considered. In a study comparing thyroid lobectomy vs. the Afirma GSC and including the costs of surveillance, Balentine et al. found that there was only a 0.3% probability of the Afirma GSC being cost-saving and a 14.9% probability of improving quality-adjusted life years [19].

Overall, the PPVs of the most common molecular tests are limited and have not sufficiently reduced the rate of unnecessary thyroid surgery (benign lesion at the final pathology report) [21]. Promising new biomarkers have been identified in the tumor microenvironment and, in particular, in the tumor immune infiltrate and could lead to significant improvements in the diagnostic performance of thyroid molecular tests in the near future [22]. Machine learning methods combining both clinical, US, and cytological data could help to better predict the risk of malignancy specific to each patient [23]. They could also integrate, in the near future, the results of the molecular tests mentioned above.

In conclusion, for Bethesda IV thyroid nodules, molecular testing can be used, if available, to supplement malignancy risk assessment in lieu of proceeding directly with diagnostic surgery. If molecular testing cannot be performed or is inconclusive, diagnostic surgery should be considered. For Bethesda class III thyroid nodules, a repeat FNAB with or without molecular testing should be considered to supplement malignancy risk assessment. If repeat FNAB, molecular testing, or both are inconclusive, either surveillance or diagnostic surgical excision may be performed, depending on clinical risk factors, US patterns, and patient preference [4,6].

### 2.4. Risk Stratification

The eighth edition of the Union for International Cancer Control (UICC) tumor, node, and metastasis (TNM) classification of malignant tumors introduced important changes for DTC [5]. Indeed, microscopic ETE now has no impact on tumor stage categories, and only gross ETE is considered in this new classification system [5]. However, although not currently used in DTC staging, recent studies have shown that microscopic ETE is still associated with a higher risk of recurrence [24]. Since the ATA risk stratification staging system was published in 2015, the criteria associated with a higher risk of persistent/recurrent disease have been revised and refined [4,5,6,7]. Some of these criteria may be evident preoperatively on physical examination or US (multifocality, gross ETE, metastatic lymph node, etc.). However, several of these factors will be available or will become apparent only after the primary treatment, with the final pathologic report (vascular invasion, extranodal extension, incomplete tumor resection, etc.), or even after the RAI adjuvant therapy (RAI-avid metastatic foci in the neck on the first post-treatment whole-body RAI scan, distant metastases). Therefore, only a small proportion of the factors known to be associated with the risk of tumor recurrence will be available preoperatively to adapt the extent of primary thyroid surgery. This situation leads, in a significant number of patients, to the need for surgical re-intervention, notably to complete the thyroidectomy [4,5,6]. 

There is a global consensus to classify in the high-risk group, patients with at least one of the following criteria: T-stage ≥ 3a, clinical N1 > 3 cm, tumor extranodal extension in metastatic lymph node(s), distant metastases. Conversely, patients with T1-2, N0 DTC without vascular invasion or aggressive histology are considered low risk or even very low risk for T1aN0 PTC by the Japan Association of Endocrine Surgeons (JAES) [4,6,7]. 

## 3. Active Surveillance for Papillary Thyroid Microcarcinoma

Recent epidemiological data have shown that the incidence of papillary thyroid microcarcinoma (PTMC) has increased substantially over the past few decades [1,3]. PTMC is defined as a PTC ≤ 10 mm in maximal diameter. The clinical significance of PTMC remains unclear, and many of them are identified incidentally after a thyroidectomy performed for other reasons [1,2,3]. Overall, the survival rates of patients with PTMC are excellent [25]. Therefore, progressively, active surveillance (AS) has become an alternative to immediate surgery for the management of low-risk PTMC, according to the guidelines published in Japan [25], the United States [6], and Korea [26].

Lee et al. performed a multicenter prospective cohort study of 1177 patients with PTMC, including 755 patients who chose AS, with a physical examination, neck US, and blood test twice a year, and 422 who underwent immediate surgery [27]. After a mean follow-up of 41 months, the PTMC progression rate (defined as an increase in tumor size >3 mm in one dimension or 2 mm in two dimensions, new ETE, or new lymph node metastasis) was 9.6% in the AS group. Baseline variables associated with the risk of disease progression under AS were age <30 years, male sex, and a tumor size of ≥6 mm [27]. In another cohort study of 1235 patients with low-risk PTMC who chose AS without immediate surgery, Ito et al. reported a progression (defined as an increase in tumor size >3 mm or new lymph node metastasis) rate of 7.1% with a mean follow-up duration of 60 months [28]. In a prospective study of AS for PTC ≤ 1.5 cm conducted in the U.S., Tuttle et al. reported a progression rate of 3.8% within 25 months of observation [29]. 

All these studies showed no fatal recurrence or disease-specific mortality in patients undergoing AS, suggesting that this approach is a safe option for the management of patients with low-risk PTMC. However, it could be argued that the duration of follow-up in these studies was insufficient to reasonably expect any cases of PTMC-related mortality. Interestingly, a QoL study using a specific assessment questionnaire (THYCA-QoL) suggested a lower QoL in the surgery group as compared with the AS group [30]. 

To date, worldwide, AS is considered an acceptable management option for low-risk PTMC [6,7]. In Japan, according to a survey in 2018, 53.8% of adult patients with low-risk PTMC were managed with AS [31]. However, many health care providers still have various concerns about AS in common clinical practice. Following a review of the literature, the JAES has clarified the potential indications for AS in patients with PTMC to facilitate its implementation [31]. In this regard, the criteria defining high-risk PTMCs that should be operated on have been identified as follows: lymph node metastasis or distant metastasis, tumor located along the course or invading the RLN, tumor adherent to or invading the trachea, aggressive subtypes of PTMC on cytology (diffuse sclerosing, solid, tall cell, columnar cell, and hobnail variants), other thyroid or parathyroid disease requiring surgery, age < 20 years. The other PTMCs can be considered low-risk and can be candidates for AS [31].

## 4. Extent of Thyroid Surgery

The extent of surgery in the initial management of DTC remains controversial. The potential higher risk of recurrence associated with less aggressive initial surgery should be balanced with the potential higher postoperative morbidity resulting from a more aggressive surgical strategy. In the context of DTC, which has a minimal impact on patient survival, preservation of QoL is always a major concern. There is still an ongoing debate regarding the appropriate extent of surgery (lobectomy vs. TT) for patients with low-risk DTC and the role of prophylactic central neck dissection (CND) for patients with a clinically negative neck (cN0). 

### 4.1. Lobectomy vs. Total Thyroidectomy for Low-Risk DTC

Whereas TT is universally recommended for the initial management of patients with T3-T4a DTC or with clinically evident neck metastasis, the appropriate surgical management of patients with low-risk DTC is much more controversial. For patients with T1b-T2, N0 DTC (tumor size between 1 and 4 cm, no ETE, and no clinical or radiographic evidence of lymph node metastases), the recommended initial surgical treatment is either thyroid lobectomy (TL) or TT [4,6,7]. In a systematic review and meta-analysis, Rodriguez Schaap et al. have shown that, for patients with low-risk DTC, similar recurrence rates (2.8 vs. 2.3%) and overall survival rates (97.4 vs. 96.8%) were achieved with TL or TT (±RAI), with a lower incidence of treatment-related complications in patients undergoing TL [32]. However, no definitive conclusion can be drawn given the retrospective observational nature of all the studies included in this meta-analysis. In a large retrospective study comprising 61,775 PTC patients from the American National Cancer Database, Adam et al. showed, after multivariable adjustment, that overall survival was similar in patients undergoing TT vs. TL for tumors between 1.0 and 4.0 cm [hazard ratio (HR) = 0.96; 95% confidence interval (CI), 0.84–1.09); *p* = 0.54] and when stratified by tumor size: 1.0–2.0 cm [HR = 1.05; 95% CI, 0.88–1.26; *p* = 0.61] and 2.1–4.0 cm [HR = 0.89; 95% CI, 0.73–1.07; *p* = 0.21] [33]. Similarly, no definitive conclusion can be drawn from this study given its retrospective nature and the short follow-up period, which is insufficient to assess the mortality of patients with low-risk PTC. Despite the substantial clinical benefits of TL with regard to surgical complications, this highlights the need for a long-term prospective clinical trial comparing the survival outcomes of TT and TL. 

As compared with TT, TL has significantly fewer side effects [32,34]. Indeed, no cases of permanent hypoparathyroidism have been reported after hemithyroidectomy [34]. In a systematic review and meta-analysis of 3827 patients, Hsiao et al. showed that patients undergoing TL had a lower risk of temporary hypoparathyroidism (2.2% vs. 21.3%; weighted RR, 0.1; 95% CI, 0.0–0.4) and of permanent hypoparathyroidism (0% vs. 1.8%; weighted RR, 0.2; 95% CI, 0.0–0.8) as compared with those undergoing TT [35]. Hypoparathyroidism after TT has been shown to be correlated with decreased overall survival, even in patients who had surgery for benign thyroid disease [34]. In a recent study analyzing 11,370 thyroid surgical procedures, Gunn et al. found, after multivariate adjustment, that RLN injury was independently associated with age ≥65 years [OR 1.6, 95% CI 1.3–2.0], TT (OR = 1.4, 95% CI 1.1–1.6), and diagnosis of thyroid malignancy (OR = 2.1, 95% CI = 1.6–2.7) (all *p* < 0.001) [36]. Similarly, in a monocentric study evaluating postoperative complications in 586 patients with PTC, Di Filippo et al. showed that TT had significantly higher rates of postoperative hypocalcaemia and RLN paralysis compared with TL (*p* < 0.001 and *p* = 0.02, respectively) [37]. Interestingly, in this study, no significant difference in the risk for locoregional recurrence or distant metastasis between TL and TT was found among patients with pT1-2 pN0 PTC [37].

A lifelong oral thyroid hormone replacement, which is systematically required in patients undergoing TT, can be avoided in approximately two-thirds of patients after TL [4,6,7]. The most robust predictive factor of hypothyroidism after hemithyroidectomy is the preoperative thyroid-stimulating hormone (TSH) level [38]. Indeed, in a recent study including 535 patients who underwent TL, Ahn et al. showed that preoperative TSH levels > 2.12 mIU/L and the coexistence of Hashimoto’s thyroiditis were significantly associated with postoperative levothyroxine supplementation [38]. The risk of levothyroxine supplementation increased by 1.401 times as preoperative TSH levels increased by 1 mIU/L [38]. In clinical practice, patients undergoing TL with preoperative TSH levels >2.5 mIU/L should be informed of the high likelihood of requiring postoperative levothyroxine supplementation, particularly in the context of a malignant thyroid nodule where high postoperative TSH levels are not recommended [4,6,38]. 

Several studies have recently investigated the impact of the type of thyroid surgery on patient QoL [39,40]. Overall, these studies found that TL was associated with improved QoL scores compared with TT and that postoperative levothyroxine supplementation was independently associated with a reduced QoL [39,40]. In addition, TT is associated with a higher risk of long-term asthenia compared with TL [41,42]. In a single institution prospective observational cohort study of 182 patients undergoing thyroid surgery, Luddy et al. found an odds ratio of asthenia for TT compared with TL of 10.4 (95% CI 3.86–28.16) [42].

The type of surgery should also be considered in the follow-up strategy for patients with DTC. Indeed, patients undergoing TT followed by RAI should have non-stimulated serum thyroglobulin (Tg) levels < 0.2 ng/mL and stimulated Tg < 1 ng/mL in the absence of interfering antibodies [4]. In patients with TL, serum Tg levels are less useful because they will not reflect the presence or absence of malignant tissue but will depend on the remaining thyroid lobe volume, current iodine status, and TSH levels [4,43]. In these patients, follow-up is performed by neck US and, when necessary, US-guided FNAB of any suspected metastatic foci [43].

Taken together, these data suggest that TL is a reliable therapeutic option for patients with low-risk DTC. Therefore, if surgery is chosen for patients with T1a (<1 cm) N0 DTC, the initial surgical procedure should be a TL unless there are clear indications to remove the contralateral lobe. TL could also be the initial standard of care for most patients with T1b (>1 cm, <2 cm) N0 DTC and for selected patients with T2N0 DTC. This conservative management approach, accepting a slightly higher risk of locoregional recurrence that will be accessible to efficient salvage therapy without impacting disease-specific survival, is a reasonable management strategy. The criteria associated with a higher risk of bilateral disease or tumor recurrence that should favor TT are as follows: radiation exposure in childhood or adolescence, family history of DTC, aggressive features on cytology, multifocality, and suspected minimal ETE on US [6,7]. The presence of other thyroid nodules in the contralateral lobe, preoperative thyroid function, and patient preferences are also important factors to consider in the decision-making process. 

### 4.2. Role of Prophylactic Central Neck Dissection

Regional lymph node metastases have been reported in up to 50% of patients with DTC. Even patients with PTMC display a significant risk of occults lymph node metastases, in particular in the central neck compartment (level VI) [44]. Metastatic lymphadenopathy is associated with increased recurrence rates and reduced long-term survival, particularly in younger patients (<45–55 years) [45,46,47]. However, microscopic nodal positivity does not carry the recurrence risk of macroscopic clinically detectable disease (cN1). There is a consensus to recommend lateral ND, as a therapeutic procedure only in patients with preoperative evidence of lymph node metastases in the lateral neck levels (cN1b) [4,6,7]. There is also a large consensus to recommend CND in patients with pre or intraoperative evidence of lymph node metastases in the central or lateral neck levels. However, while there is no indication for prophylactic lateral ND (in the setting of cN0 DTC), the role of prophylactic CND (pCND) is still controversial [46,47]. Indeed, surgical practices regarding the use of pCND differ largely between centers of care and countries, with also different recommendations according to learned societies [4,6,7].

There are several systematic reviews with contradictory results on the role of pCND in patients with cN0 DTC. Definitive conclusions are difficult to draw due to a significant heterogeneity in inclusion criteria, tumor size, and therapeutic interventions [46,47,48,49,50,51,52,53]. There is no evidence of any benefit in terms of overall survival from pCND in low-risk DTC patients [53]. In a recent systematic review and meta-analysis of 18,376 patients with cN0 PTC, Chen et al. found that pCND was associated with significantly lower locoregional recurrence rates (OR 0.65; 95% CI 0.48–0.88) but significantly higher incidence rates of transient RLN injury (OR 2.03; 95% CI 1.32–3.13), transient hypocalcemia (OR 2.23; 95% CI 1.84–2.70), and permanent hypocalcemia (OR 2.22; 95% CI 1.58–3.13) than absence of pCND [8]. Similar results were reported in another meta-analysis on 6930 cN0 PTC patients reported by Zhao et al. showing that compared with TT alone, TT + pCND significantly reduced the risk of locoregional recurrence but increased the incidence rates of temporary and permanent hypoparathyroidism and temporary RLN palsy [51]. 

Proponents of routine pCND argue that it identifies occult metastatic lymph nodes in the central neck in approximately one third of patients with clinical T1-T2N0 DTC and that this finding can serve to adapt the therapeutic management of patients [44,46,47]. The proportion of patients with occult central lymph node metastases can be higher in more advanced T-stage categories (T3 or T4). Indeed, Hughes et al. reported an occult positivity rate of 62% in 78 patients with DTC > 1 cm undergoing TT with pCND [45]. In a retrospective analysis of 49 patients with DTC who underwent TT + pCND, Wang et al. showed that pCND resulted in the detection of unsuspected metastatic lymphadenopathy in 41% of patients and changed RAI recommendations in 14% of patients [46]. As mentioned above, decreased locoregional and central compartment recurrence rates have been shown with pCND [46,47]. Moreover, salvage surgery for metastatic lymph node recurrences in the central neck is known to be difficult and associated with an increased risk of RLN injury compared with primary surgery [54]. In a comprehensive review regarding the management of locoregional recurrent DTC, Cavalheiro et al. reported RLN permanent paralysis rates ranging from 0% to 12%, whereas this risk does not exceed 2% for primary surgery [54]. Another potential benefit of performing a pCND is its ability to improve post-treatment biochemical cure rates (undetectable Tg levels). Indeed, some studies reported lower post-treatment Tg levels with pCND + TT compared with TT alone [45,48,49]. Indeed, in a series of 447 patients with cN0 PTC undergoing TT ± CND, Sywak et al. showed that the rate of patients with undetectable postoperative Tg levels was significantly higher in the TT + CND group compared with the TT alone group (72% vs. 43%; *p* < 0.001) [48]. 

On the other hand, the main argument against performing pCND in patients with cN0 DTC is that these patients display excellent survival outcomes when managed with thyroidectomy without CND [4,8,53]. Although pCND can improve locoregional control and recurrence-free survival, it has definitively no positive impact on overall survival, whereas it increases postoperative complication rates and particularly the risk of permanent hypoparathyroidism [4,6,8,44,53]. In a retrospective review of 1129 PTC patients who had TT ± CND, Nixon et al. showed a 10-year disease-specific survival of 100% in the 275 patients who underwent TT without pCND [50]. The rate of structural recurrence in the central neck was 0.4% (1/275), and the rate of reoperation in the central neck was 0% [50]. Indeed, the risk of central neck recurrence in low-risk DTC patients treated without pCND is considerably lower than the rate of occult level VI node metastases in this population. This suggests that the majority of microscopic metastatic lymph nodes will not progress despite the absence of any additional treatment. 

Taken together, these data indicate that pCND should be considered in patients with advanced primary tumors (T3 or T4) since they display a significant risk of locoregional recurrence that could be lowered by a pCND. Thyroidectomy (TL or TT) without pCND is appropriate for cN0 T1-T2 PTC. However, in these patients, ipsilateral pCND is also a reasonable option if the histological information provided by pCND can be used to refine the prognosis and guide subsequent treatment and follow-up. Given their lower risk of node metastasis, thyroidectomy without pCND is recommended for most low-risk follicular carcinomas. 

## 5. Innovative Technological Tools in Thyroid Surgery

### 5.1. Hemostasis Energy Devices

Thyroid surgery has long been associated with a high risk of postoperative bleeding and hematoma. After TT, a critical airway compromise secondary to wound hematoma represents a life-threatening complication. Refinements in surgical techniques have considerably minimized the risk of postoperative bleeding. Indeed, in recent studies, the risk of postoperative hematoma after thyroid surgery was under 1% [55,56]. Progressively, systematic wound drainage has been discontinued, and the postoperative length of stay has been considerably reduced [55].

Since the early 2000s, new hemostasis energy devices (Harmonic, Ligasure, Thunderbeat devices, etc.) have been developed [56,57,58,59]. These devices represent a safe and efficient alternative to the traditional clamp-and-tie hand technique in thyroid surgery, yielding a reduction in operating time while not increasing RLN injury rates [56,57,58,59]. The sealing of vessels up to 7 mm in diameter can be securely achieved with these devices, which is largely sufficient for thyroid surgery [59]. Although thermal spread is minimal, these devices should be used with caution near the RLN, particularly at its laryngeal penetration. A reduction in the risk of postoperative hematoma following thyroid surgery has been shown with these devices compared with conventional hemostatic techniques [56,57]. There is no clear evidence that one device is superior to another for thyroid surgery [57,58,59]. Each surgeon may have his own preferences according to his experience and skills, but surgical morbidity in thyroid surgery remains highly associated with surgical volumes [4,6,55].

### 5.2. Intraoperative Nerve Monitoring

Intraoperative nerve monitoring (IONM) has been introduced in thyroid surgery to assist the surgeon in RLN identification and dissection. IONM can efficiently predict postoperative RLN function. The IONM systems use electromyography of the vocal cords to monitor the electrophysiological activity of the RLNs. This method of neuromonitoring is performed intermittently through ipsilateral stimulation of the RLN using a handheld monopolar or bipolar probe [60]. Changes in the pattern of the signal may indicate RLN irritation and a possible loss of function. 

Despite the widespread adoption of IONM into common surgical practice, many studies showed no significant reduction in RLN injury rates, particularly when surgery is performed by high-volume surgeons [60,61,62,63]. For example, in their meta-analysis of 23,512 patients (35,513 nerves at risk; NAR), Pisanu et al. demonstrated no advantage for IONM in reducing RLN injury compared with visualization alone (3.47% vs. 3.67%, respectively) [61]. The drawback of initial IONM systems was the risk of RLN injury between intermittent stimulations; this technology was only able to detect possible RLN damage post hoc. To work around this problem, continuous IONM systems using a temporary implantable electrode attached to the vagus (X) nerve have been developed to allow uninterrupted laryngeal monitoring [64]. A recent non-comparative meta-analysis suggested that this technology was safe and effective to preserve RLN, with reported transient and permanent RLN palsy of 2.26% and 0.05%, respectively [64]. Only one case of transient vagus nerve palsy (electrode dislodgement) and one case of hemodynamic instability were observed [64]. However, more data are still needed to support the use of this technology in thyroid surgery. 

IONM helps to determine the exact location of RLN injuries and identify segments of neuropraxia [65]. This makes it possible to minimize the risk of bilateral RLN by delaying resection of the controlateral lobe when RLN injury is suspected after TL. In this regard, the risk of false-positive losses of signal due to technical issues should be weighed against the risk of bilateral RLN palsy if the loss of signal is real and a second RLN injury occurs [66,67]. A cost-effectiveness study, published in 2017, found that the use of IONM was cost-effective in avoidance of bilateral RLN injury in patients undergoing TT [67]. 

IONM could also be used to assess the function of the external branch of the superior laryngeal nerve (EBSLN), which innervates the cricothyroid muscle to promote lengthening and thinning the vocal fold, thus increasing voice pitch [68]. Injury of the EBSLN, which mainly occurs when ligating the upper pole of each thyroid lobe, is a common cause of dysphonia after thyroid surgery, despite the absence of RLN palsy. In a prospective study of 176 consecutive nerves at risk, Del Rio et al. showed that intraoperative recognition and stimulation of the EBSLN, performed before any dissection of the superior vascular thyroid pole, led to a much higher rate of nerve conservation [68]. However, more data are still needed to definitively support this hypothesis.

### 5.3. Identification of Parathyroid Glands

Intra-operative identification and preservation of parathyroid glands is an important but challenging aspect of thyroid surgery. Parathyroid glands could be injured or accidentally removed during thyroid surgery, which can lead to transient or permanent hypocalcemia [69,70]. Currently, intra-operative identification of parathyroid glands is achieved through direct visual inspection and is largely dependent on surgical experience. Thyroid surgery for malignant disease and performing CND are associated with a higher risk of permanent hypoparathyroidism [44,70]. In this context, several technologies have been developed to facilitate identification and preservation of parathyroid glands, such as Raman spectroscopy, carbon nanoparticle injection, shear wave elastography, laser speckle contrast imaging, dynamic optical contrast imaging, indocyanine green (ICG) angiography, and near-infrared-induced autofluorescence (NIRAF) [71]. 

ICG angiography is the most studied method and is considered to be a reliable aid in identifying parathyroid glands [71]. ICG is a non-toxic, near-infrared exogenous fluorescent agent that binds to plasma proteins and becomes illuminated once exposed to near-infrared light at a wavelength of 806 nm [71,72]. ICG is a non-selective agent and does not specifically target parathyroid tissues. However, parathyroid glands receive a higher amount of blood flow compared with surrounding tissues and thus emit a stronger contrast signal. ICG is injected following retraction of the thyroid lobe, and near-infrared light is used to detect a fluorescent signal from the parathyroid glands. It can also be re-injected following TL to assess parathyroid gland perfusion [71,72]. In a recent literature review, Spartalis et al. considered that intra-operative ICG angiography was a simple, rapid, and reproducible method facilitating visualization of parathyroid glands intraoperatively that could assist surgeons in their decision-making [72]. However, the ICG fluorescence imaging technique for detection of parathyroid glands still lacks standardization, and further studies are needed to establish its clinical utility [71,72]. 

Autofluorescence is defined as the natural emission of light from tissues when their biological substrates (endogenous fluorophores) are exposed to radiation of a suitable wavelength without the need for exogenous agents such as ICG. Parathyroid glands were found to spontaneously emit light in the infrared spectrum with a fluorescence peak at about 820 nm when exposed to near-infrared light, which is 11 times higher than the surrounding tissue [71,73]. Studies utilizing different types of NIRAF technology have shown promising results [73,74]. A device consisting of a portable spectrometer, a 785 nm diode laser, and a 2 mm optical fiber was able to successfully identify 97% of parathyroid glands among 137 patients who underwent thyroidectomy and/or parathyroidectomy [75]. Several studies used the Fluobeam 800 system, where a laser provides radiation at 750 nm and collects the optical signal for wavelengths above 800 nm [74,75,76]. The percentage of identified parathyroid glands ranged between 76.3 and 100% [74,75,76]. A recent meta-analysis found a sensitivity of 0.98, a specificity of 0.99, and an area under the curve of 0.99 in the identification of parathyroid glands using NIRAF systems in thyroid surgery [77]. These encouraging results are supported by a randomized clinical trial of 241 patients undergoing TT showing that the use of NIRAF lowered the temporary postoperative hypocalcemia rate from 22% to 9% (*p* = 0.007), the parathyroid auto-transplantation rate from 13% to 3% (*p* = 0.009), and the parathyroid inadvertent resection rate from and 12% to 2.5% (*p* = 0.006) [78]. The reliability of NIRAF technologies is currently limited by their difficulty in locating parathyroid glands covered by other tissues, as well as by false-positive cases due to brown fat, colloid nodules, metastatic lymph nodes, and bloodstaining in the operative field [77,78]. 

NIRAF is therefore an emerging tool that facilitates intra-operative parathyroid gland identification and reduces the rate of post-operative hypocalcaemia in a safe and reproducible manner. Prospective randomized controlled trials are needed to evaluate the real-life impact of NIRAF technologies on the clinical outcomes of patients undergoing thyroid surgery. 

### 5.4. Innovative Surgical Approaches

**Figure 1:**
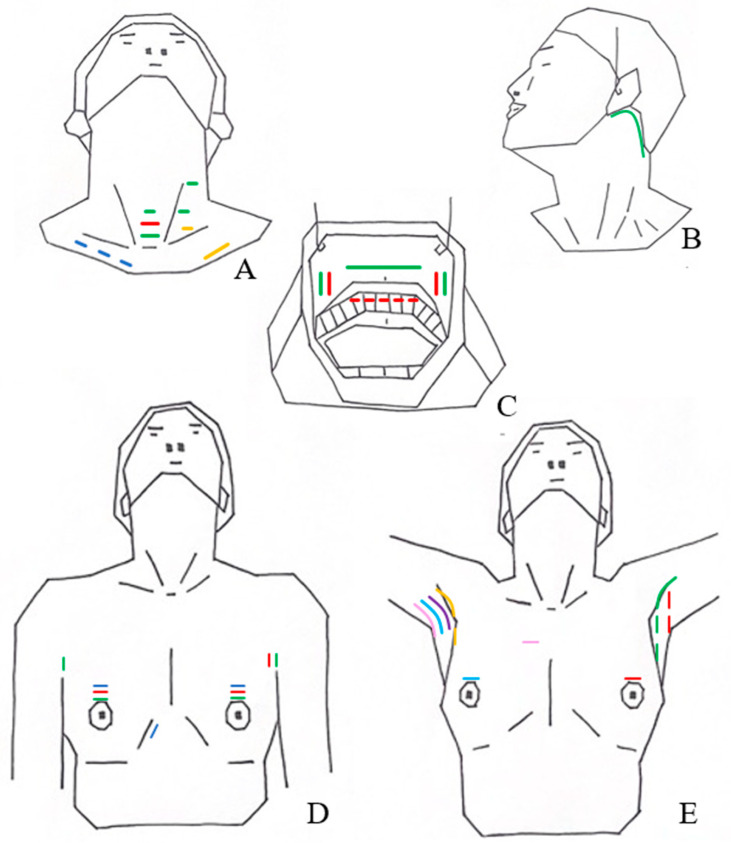
Design of skin incisions in various types of robotic/endoscopic thyroidectomies. (**A**) – Cervical approach with CO_2_ insufflation; – Minimally invasive video-assisted thyroidectomy; – Anterior chest approach with CO_2_ insufflation; – Video-assisted neck surgery; (**B**) – Postauricular facelift approach; (**C**) – Transoral sublingual and vestibular approach with CO_2_ insufflation; – Transoral vestibular approach with CO_2_ insufflation; (**D**) – Bilateral axillo-breast approach with CO_2_ insufflation; – Axillo-bilateral breast approach with CO_2_ insufflation; – Breast approach with CO_2_ insufflation; (**E**) – Axillary approach with CO_2_ insufflation; – Unilateral axillo-breast approach with CO_2_ insufflation; – Gasless unilateral axillary approach; – Single-incision transaxillary approach; – Gasless unilateral axillo-breast approach; – Gasless transaxillary approach with anterior chest port.

The original approach to thyroidectomy was through a large (8–10 cm) collar incision. Surgical techniques have evolved, and currently, open thyroidectomies are performed generally through a 4–6 cm incision made in the anterior lower neck. Over the past 20 years, alternative approaches have been developed, including minimally invasive video-assisted endoscopic approaches and remote access surgery, to minimize surgical morbidity and improve aesthetic outcomes of thyroid surgery [79,80]. Robotic and endoscopic surgical approaches can be classified according to the use of carbon dioxide (CO_2_) gas insufflation and the site of incision and are summarized in Table 2 [79,80]. Their corresponding surgical incisions are shown in Figure 1. The most commonly employed approaches are the bilateral axillo-breast approach (BABA) and the gasless transaxillary, retroauricular facelift, and transoral vestibular approaches [79,80,81,82,83,84]. 

The indications for endoscopic/robotic thyroidectomy may include benign thyroid nodules or follicular neoplasms less than 6 cm in diameter and DTC less than 4 cm without gross ETE extension [79,80,85]. CND can be performed through the same surgical approaches [85,86]. Exclusion criteria include gross ETE, lymph node metastasis with invasion of surrounding structures, large substernal goiters, and a history of neck irradiation or surgery. Grave disease or Hashimoto thyroiditis could be relative contraindications due to an increased risk of bleeding [79,85,86]. 

Each of the four most common robotic approaches has its own advantages and drawbacks. They are compared in Table 3 [80,81,82,83,84,85].

Remote access thyroidectomy has many advantages, such as hidden scars and an enlarged surgical view. In particular, the use of robotics allows a three-dimensional view of the operating field, a greater degree of movement, and the elimination of hand tremors [79,80]. However, remote access thyroidectomy has several drawbacks, such as a wide skin flap elevation to create work space and a wider dissection area to reach the thyroid gland [79,80]. TT could be difficult with gasless unilateral transaxillary and facelift approaches [79,80,81,82]. The other most commonly accepted limitations of robotic thyroid surgery are a longer operative time and a long learning curve (approximately 40 to 45 cases) [87]. Moreover, the prohibitive cost of robot-assisted thyroid surgery, related both to the medical equipment costs and the longer operative time, is a major barrier to widespread use of the technique [85,88]. 

Regarding safety, in several meta-analyses, rates of complications such as hypoparathyroidism and RLN injuries were not significantly different in robotic vs. open-access thyroidectomy [80,88,89]. However, in subgroup analyses, RLN injury was more frequent early in the learning curve and with low-volume surgeons, which underlines the importance of an appropriate training program [89,90]. Unusual complications can occur, such as transient brachial plexus injury, with the robotic transaxillary approach [87,89]. Transient dysesthesia in the distribution of the greater auricular is universal in the postauricular facelift approach [81,82]. Anterior chest transient paresthesia is common after BABA thyroidectomy [84]. Mental nerve injury can occur with the transoral vestibular approach [83,86]. Serious CO_2_ embolisms have been reported in procedures using CO_2_ insufflation [87,89]. 

There has been concern regarding oncologic outcomes with these innovative surgical approaches. In different meta-analyses, there were, however, no significant differences regarding oncologic outcomes between remote access thyroidectomy and conventional open thyroidectomy [91,92,93]. Further studies with long-term follow-up and large patient samples are needed to confirm these results. 

Cosmetic excellence is the most important reason for patients and surgeons to choose remote access thyroidectomy [92,94]. Indeed, the cosmetic outcome is superior with remote access compared with conventional thyroidectomy [94]. However, the impact of culture on cosmetic outcomes is essential. In Asia, the social impact of visible neck scars is high, which endorses extracervical approaches [95]. In contrast, in North America and Europe, there is little concern with cervical scars. Indeed, the cosmetic outcomes of conventional open thyroidectomy are well accepted in most western countries [94,95]. It is therefore likely that cultural perceptions regarding cervical scars have a great influence on the use of extracervical approaches to thyroid surgery. 

All together, these data suggest that conventional open thyroidectomy is still the standard surgical approach for most patients with DTC. However, remote access thyroidectomy is a viable alternative, when performed by high-volume surgeons, for selected patients with particular concerns regarding neck scars.

### 5.5. Outpatient Thyroidectomy

Thyroidectomy has traditionally been considered an inpatient procedure owing to concerns over the potential life-threatening consequences of a compressive postoperative wound hematoma [55,56]. However, recent years have seen an increase in the volume of outpatient thyroidectomy because the risk of postoperative hematoma has become extremely low for high-volume thyroid surgeons [55,56]. Postoperative hypocalcemia is also a potential concern in patients undergoing TT or completion thyroidectomy since it can occur after patient discharge [69]. TL has been safely performed as an outpatient procedure in many centers because, contrary to TT, the risk of tracheal compression due to a potential wound hematoma is almost absent. However, outpatient TT is still a controversial procedure, and many surgeons still discharge the patient on day 1 after surgery [55]. 

The 2015 ATA guidelines stipulated that outpatient thyroidectomy could be undertaken safely in a carefully selected patient population with certain precautionary measures [4]. Thereafter, two meta-analyses have confirmed that outpatient thyroidectomy could be safely performed by an experienced surgeon with adequate infrastructure and good patient selection [96,97]. In most studies, patient selection criteria include no major medical comorbidities, social considerations (cognitive ability, patient and caregiver preoperative education, patient caregiver accessibility, proximity to a skilled facility, etc.), or clinical features (tumor size, clinical presentation, type of procedure, etc.) [96,97]. To manage the risk of postoperative hypocalcemia, some surgeons prescribe a systematic calcium supplementation or use the postanesthesia care unit’s rapid parathyroid hormone (PTH) dosage as a major discharge criterion [98].

Therefore, careful patient selection and a systematized protocol are needed before incorporation into clinical practice of outpatient thyroidectomy, particularly for patients undergoing TT.

## 6. Conclusions 

Surgery has a central role in the multidisciplinary management of patients with DTC. Despite several international therapeutic guidelines, there are still ongoing debates on the surgical management of DTC. Appropriate selection of patients who are candidates for thyroid surgery is essential and involves, in addition to conventional clinical, US, and FNAB data, the results of molecular testing. 

The role of AS in the management of patients with PTMC has been progressively well defined. Conservative surgical procedures such as TL have become the standard of care for most patients with low-risk DTC. The role of pCND is still highly controversial for patients with cN0 DTC, although the pathological information provided by pCND can assist in establishing the indication for further treatments (completion thyroidectomy, RAI). Minimally invasive techniques have been developed for thyroid surgery, and remote access thyroid surgery may be considered by high-volume surgeons when there are specific concerns regarding neck scars. 

Randomized prospective studies could clarify the role of all these surgical advances when contradictory data precludes clear and definitive conclusions. Finally, an optimal thyroid surgery for patients with DTC should be an integral part of multidisciplinary management and should be based on comprehensive patient information and consent.

## Figures and Tables

**Table 1:** Characteristics of different thyroid molecular tests.

Test Characteristics	ThyroSeq	Afirma GSC	ThyraMIR
**Molecular test**	NGS	RNA sequencing	microRNA analysis
**NPV**	High	High	Scant data
**PPV**	Intermediate	Low	Scant data
**Sensitivity/specificity**	High/High	High/Intermediate	High/High
**Main relevance**	Rule-in test	Rule-out test	Rule-in and rule-out test
**Data analysis**	Centralized or local labs	Centralized labs	Local labs

NGS: next-generation sequencing; NPV: negative predictive value; PPV: positive predictive value; high, intermediate and low values were defined by values >70%, between 60 and 70% and <70%, respectively.

**Table 2:** Classification of robotic and endoscopic thyroidectomies.

Methods with CO_2_ Insufflation	Gasless Methods
Cervical approach	Minimally invasive video-assisted thyroidectomy (MIVAT)
Anterior chest approach	Anterior chest approach
Axillary approach	Video-assisted neck surgery
Breast approach with parasternal port	Axillary approach
Axillo-breast approach	Axillary approach with anterior chest port
Axillo-bilateral breast approach (ABBA)	Single incision axillary approach
Bilateral axillo-breast approach (BABA)	Gasless unilateral axillo-breast or axillary approach
Unilateral/bilateral axillo-breast approach	Facelift (retroauricular) approach
Transoral approach	Transoral approach

**Table 3:** Comparison of the four most common types of robotic thyroidectomies.

Surgical Characteristics	Gasless Axillary	BABA	Gasless Facelift	Transoral
Difficulty creating the WS	++++	++++	+++	++
Manipulability of instruments in the WS	++++	+++	+++	+++
Clarity of surgical view	++++	+++	++++	+++
Applicability of TT	+	+++	+/−	+++
Applicability of CND	+++	++	+++	+++
Applicability of LND	++++	++	++++	+/−

BABA: bilateral axillo-breast approach; WS: working space; TT: total thyroidectomy; CND: central neck dissection; LND: lateral neck dissection; The fit between each surgical approach and each surgical characteristic is ranked from +/− (low) to ++++ (very high)
